# The Effect of Conservative vs. Radical Treatment of Ameloblastoma on Recurrence Rate and Quality of Life: An Umbrella Review

**DOI:** 10.3390/jcm13175339

**Published:** 2024-09-09

**Authors:** Roberta Gasparro, Francesco Giordano, Maria Domenica Campana, Angelo Aliberti, Elena Landolfo, Pasquale Dolce, Gilberto Sammartino, Alessandro E. di Lauro

**Affiliations:** 1 Department of Neurosciences, Reproductive Sciences and Oral Sciences, Section of Oral Surgery, University of Naples Federico II, 80131 Naples, Italy; roberta.gasparro@unina.it (R.G.); mariadomenica.campana@unina.it (M.D.C.); angelo.aliberti3@gmail.com (A.A.); e.landolfo@studenti.unina.it (E.L.); alessandroespedito.dilauro@unina.it (A.E.d.L.); 2 Department of Medicine, Surgery and Dentistry, University of Salerno, 84081 Salerno, Italy; frgiordano@unisa.it; 3 Department of Translational Medical Sciences, University of Naples Federico II, 80131 Naples, Italy; pasquale.dolce@unina.it

**Keywords:** ameloblastoma, radical treatment, conservative treatment, recurrence, clinical management

## Abstract

Ameloblastoma is a rare, benign, but locally aggressive odontogenic tumor that originates from the epithelial cells involved in tooth development. The surgical approach to treating an ameloblastoma depends on the type, size, location, and extent of the tumor, as well as the patient’s age and overall health. This umbrella review’s aim is to summarize the findings from systematic reviews (SRs) and meta-analyses on the effect of radical or conservative treatment of ameloblastoma on the recurrence rate and quality of life, to evaluate the methodological quality of the included SRs and discuss the clinical management. Three electronic databases (PubMed, Scopus, The Cochrane Library) were checked. The primary outcome was the recurrence rate after surgical treatment, while the secondary outcomes were the post-operative complications, quality of life, esthetic, and functional impairment. The methodological quality of the included SRs was assessed using the updated version of “A Measurement Tool to Assess Systematic Review” (AMSTAR-2). Eighteen SRs were included. The quality of the included reviews ranged from critically low (three studies) to high (eight studies). Four studies were included in meta-analysis, and they revealed that the recurrence rate is about three-times more likely in the conservative treatment group compared to the radical treatment group, and this result is statistically significant. Despite the high recurrence rate, the latter was more appropriate in the case of smaller lesions and younger patients, due to better post-operative quality of life and reduced functional and esthetic impairments. Based on the results of this overview, conservative treatment may be recommended as the first-line approach for intraosseous ameloblastoma not involving soft tissue. However, given the expectation of a higher recurrence rate, it is advisable to reduce the interval between follow-up visits. However, further prospective studies are needed to establish the best treatment choice and follow-up period.

## 1. Introduction

Ameloblastoma is a rare, benign odontogenic tumor of epithelial origin, accounting for approximately 10% of all jaw tumors [1] and 13–58% of all odontogenic tumors [2]. The global incidence rate of ameloblastoma is 0.92 per million population per year, with heterogeneous incidence rates between studies [3]. Among all the cases, 53.2% cases are male and 46.7% are female, with a male/female ratio of 1.14:1. Overall, the peak incidence of ameloblastoma, worldwide, is in the third decade [3]. Despite its benign nature, ameloblastoma exhibits locally invasive growth, rare metastases, and has high rate of recurrence [4], posing significant challenges in clinical management and impacting patient quality of life and healthcare systems, requiring substantial resources for surgical interventions, long-term follow-up, rehabilitation, and ongoing care [5,6]. The World Health Organization (WHO) 2022 classification of ameloblastoma reflects the latest understanding of its diagnosis, histopathological features, and clinical behavior [7]. Three types of ameloblastoma have been described. Conventional ameloblastoma, previously known as solid/multicystic ameloblastoma, is the most common type, typically occurring in the mandible and exhibiting various histopathological patterns, including follicular, plexiform, acanthomatous, and desmoplastic [8,9]. Unicystic ameloblastoma is a cystic lesion that, while presenting clinical and radiological characteristics typical of an ordinary jaw cyst, contains ameloblastomatous cells within the epithelial lining of the cyst upon histological examination. These cells may or may not infiltrate the lumen of the cyst or its connective tissue wall. This type accounts for approximately 5 to 22% of all ameloblastomas, primarily affecting younger individuals, and presents three histological variants: luminal, intraluminal, and mural [10,11]. Peripheral ameloblastoma is a rare variant occurring in the soft tissues overlying the jaws. Typically, it is less aggressive than intraosseous forms [12,13].

The most common symptom of ameloblastoma is a painless swelling or expansion of the jaw, typically affecting the mandible more than the maxilla [14]. This swelling can become noticeable over time as the tumor grows. Due to the swelling and expansion, patients may exhibit noticeable facial asymmetry. Although often painless initially, as the tumor enlarges, it can cause pain or discomfort, particularly if it invades surrounding tissues or structures, with possible tooth displacement and mobility [15].

Conventional ameloblastoma may appear multilocular (“Soap Bubble” or “Honeycomb”), and this is the classic presentation, where the lesion appears as a radiolucent area with multiple internal septations, creating a bubble-like pattern, or as unilocular radiolucency. In some cases, particularly in smaller or early-stage lesions, ameloblastomas may present as a single, well-defined radiolucent area [16].

The differential diagnosis of ameloblastoma may be difficult when lesions and tumors of the jaw can present similar clinical and radiographic findings. The main conditions to consider are the Odontogenic Keratocyst [17], Dentigerous Cyst, Adenomatoid Odontogenic Tumor (AOT), and Central Giant Cell Granuloma (CGCG) [18].

Currently, surgery is considered the most effective therapeutic option for this odontogenic lesion. To achieve complete excision of the lesion, either a conservative or radical approach can be employed for the treatment of ameloblastoma.

Although invasive surgical procedures like enucleation and resection are commonly preferred treatments, they can lead to serious complications, such as facial deformities, maxillary bone fractures, dental losses, and paresthesia [19,20,21]. In this regard, more conservative surgical techniques, such as marsupialization and decompression, may be suitable options [22]. These techniques are significantly less invasive, and several studies have reported positive results in reducing jaw lesions [23].

Despite the prevalence of surgical intervention, the optimal treatment approach for ameloblastoma remains debated, with various systematic reviews (SRs) examining outcomes like recurrence rates, quality of life, and esthetic and functional impairment. Given the existing body of SRs, an overview of SRs is warranted to synthesize the available evidence, assess the quality of the SRs, and provide clinicians with a comprehensive summary. To our knowledge, this is the first overview conducted on this topic. This overview aims to summarize the findings from SRs and meta-analyses on patients with primary or recurrent conventional or unicystic ameloblastoma treated with radical and conservative approaches, evaluate the methodological quality of the included SRs, and discuss the clinical management of this complex oral pathology.

## 2. Materials and Methods

This review was designed as an umbrella review (overview of systematic review) with a meta-analysis. It was compiled adhering to the PRISMA (Preferred Reporting Items for Systematic Reviews and Meta-Analyses) guidelines. According to the PICO (P: population, I: intervention, C: comparison, O: outcome) protocol, this overview aimed to answer to the following question: “Does conservative surgical treatment of ameloblastoma (intervention) lead to a higher recurrence rate (outcome) according to patient’s age and dimension of tumor, compared to radical surgical treatments (Comparison), in patients with primary ameloblastoma or with a recurrent ameloblastoma (Population)?” Conventional surgical treatments are considered as enucleation, curettage, peripheral ostectomy, marsupialization, decompression, Carnoy’s solution or a combination of these techniques, while invasive surgical treatments are considered segmental resection, marginal resection, emimandibulectomy/emimaxillectomy, total jaw resection. All histological types of ameloblastoma were included. Ameloblastic carcinoma and metastasizing ameloblastoma were excluded. The primary outcome was the recurrence rate after surgical treatment, while secondary outcomes were the post-operative complications, quality of life, esthetic, and functional impairment (functional limitations in chewing, speaking, sleeping and inability to perform daily routines and work activities correctly).

### 2.1. Literature Search

Initially, a pilot search was conducted on PubMed to check the presence of existing overviews and enough systematic reviews (SRs) that could serve as a solid foundation for the creation of the above-mentioned overview. Literature research was conducted for reviews and meta-analyses published up to June 2024 using three electronic databases (PubMed, Scopus, The Cochrane Library). Different combinations of keywords and MeSH terms, according to the database’s rules, were developed to identify suitable studies. Search strategy is reported in Table 1.

A manual search was performed in oral surgery journals (International Journal of Oral and Maxillofacial Surgery, Oral Disease, Japanese Dental Science Review), and a further search was performed among the references of the included articles. An attempt to explore grey literature involved searching through conference abstracts published on Web of Science and Scopus, as well as databases of scientific dental congresses (Società Italiana di Chirurgia Odontostomatologica (SIdCO), International Association for Dental Research (IADR), Società Italiana di Patologia e Medicina Orale (SIPMO), European Association of Oral Medicine (EAOM)). Moreover, the reference lists of all included studies and relevant review articles were manually examined to identify any additional studies that may have been missed during the electronic search. The review’s selection was performed by two independent reviewers (MDC, EL). Eligibility criteria were only SRs and meta-analyses addressing the recurrence rate of ameloblastoma and quality of life after a conventional or radical surgical treatment, in English language, published up to June 2024. The exclusion criteria were as follows: clinical controlled trials (CCTs) and randomized controlled trials (RCTs), duplicate publications, narrative reviews, case series, surveys, radiographic studies, studies with solely histological data, animal studies, case reports, letters to the editor, and in vitro studies. Additionally, abstracts and articles written in languages other than English were excluded. Following the screening of titles and abstracts, articles were selected for full-text eligibility. In cases where discrepancies arose in assessing the eligibility of titles and abstracts, full texts were included for final evaluation. Any disagreements between the two reviewers were resolved through the involvement of a third reviewer (GS).

Potential sources of bias like selection bias, publication bias, and heterogeneity of included reviews were addressed and minimized by using a thorough and systematic search strategy, clearly reporting the selection criteria for included reviews and evaluating the methodological quality of each systematic review with tools like AMSTAR-2.

### 2.2. Data Extraction

Two authors (MDC, EL) independently extracted data using a pre-established extraction form to minimize the risk of errors and bias. Each reviewer recorded the data on a separate extraction form. In cases where clarity was lacking in the systematic reviews (SRs), the individual studies themselves were consulted. No further details were sought from the authors. After independent extraction, the two reviewers compared their forms to identify discrepancies. Any differences were discussed and resolved through consensus. If consensus could not be reached, a third reviewer was consulted. From each study, author, publication year, search period, databases, study design (SR with or without meta-analysis), diagnosis, intervention and control groups, quality tool and quality of the individual studies, outcome measures, results, and author’s conclusion were extracted.

### 2.3. Methodological Quality of Included Reviews

The methodological quality of the included SRs was independently assessed by two reviewers [VS, AA] using the updated version of A Measurement Tool to Assess Systematic Review (AMSTAR-2) [24]. This independent assessment helped minimize bias and ensured that all aspects of the review were thoroughly evaluated. AMSTAR-2 is a valid and reliable instrument made of 16 items (Protocol Registration, Literature Search Adequacy, Study Design Criteria, Search Strategy Details, Study Selection Process, Data Extraction Process, Explanation of Exclusions, Description of Included Studies: Risk of Bias Assessment, Funding Source Disclosure, Meta-Analysis Methods, Impact of Bias on Results, Risk of Bias in Interpretation, Heterogeneity Assessment, Statistical Methods, Conflicts of Interest), which correspond to three possible responses: “yes,” (indicating the criterion was met), “partial yes” (partially met), or “no.” (not met). Following the assessment of weaknesses identified in both critical and non-critical aspects, the overall quality rating of a systematic review (SR) was categorized as “high”, “moderate”, “low”, or “critically low” as follows: high: no or one non-critical weakness; moderate: more than one non-critical weakness; low: one critical flaw with or without non-critical weaknesses; critically low: more than one critical flaw with or without non-critical weaknesses.

### 2.4. Statistical Analysis

Statistical analysis was conducted using the Restricted Maximum Likelihood method. The pooled effect size was reported with a 95% confidence interval (CI). Heterogeneity was assessed using the I^2^ statistic, and a 95% prediction interval (PI) was calculated. An I^2^ value greater than 50% indicated significant heterogeneity, while the 95% PI estimated the potential range of true effects for future studies. Publication bias was assessed using Egger’s regression test. Additionally, a test for excess significance was performed to determine whether the observed number of statistically significant results exceeded the expected number, suggesting potential data tortures or reporting bias. This assessment was conducted using the Proportion of Statistical Significance Test (PSST). If a study was included in multiple meta-analyses, only one instance was retained to avoid bias. Multiple effect sizes reported for a single study were retained if they originated from independent subgroups. In cases where multiple studies shared participants from the same group but compared them to different groups, these studies were identified, and adjustments were made to the calculations by dividing the shared sample size by the number of studies using it.

All statistical analyses were performed using the ‘metaumbrella’ package in the R statistical software (version 4.3.3) [25]. Statistical significance for all tests was set at α = 0.05.

## 3. Results

### 3.1. Search Results

**Figure 1:**
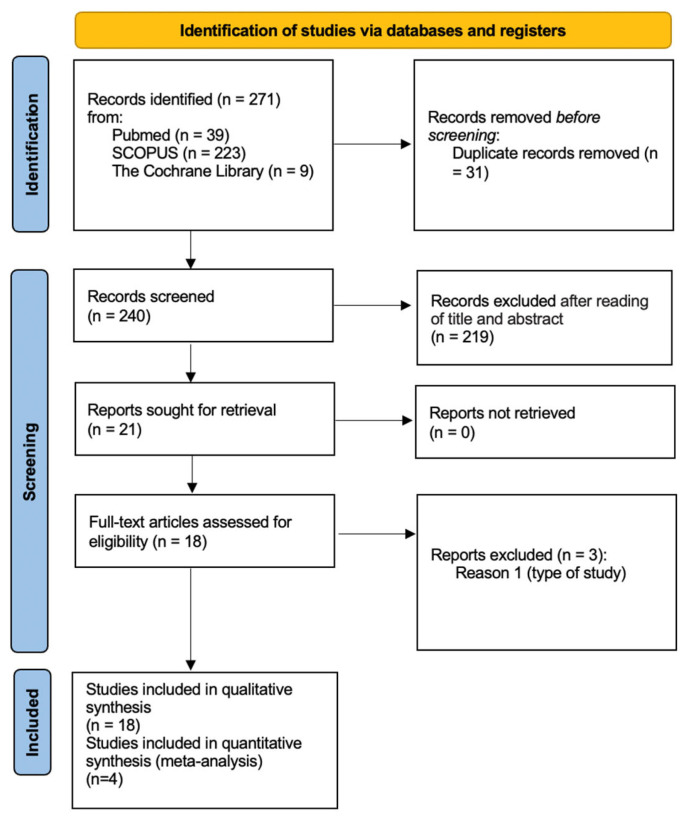
**PRISMA diagram.** From: https://www.bmj.com/content/372/bmj.n71 (accessed on 7 September 2024).

Figure 1 shows a flow diagram of the study selection.

Thus, 271 records were discovered through both electronic and manual searches. Following the removal of duplicates, the titles and abstracts of 240 records were reviewed. Of these, 56 articles were included for full-text reading, while 29 were excluded according to the application of the exclusion criteria.

Finally, 18 SRs were included for the qualitative analysis [26,27,28,29,30,31,32,33,34,35,36,37,38,39,40,41,42,43].

### 3.2. Characteristics of Included Reviews

Data extracted from the eighteen (18) SRs are summarized in Table 2. The number of primary studies included in each SR ranged between 6 and 76. Some of SRs were integrated with a meta-analysis [26,29,33,34,38,39,41,42]. Most of the systematic reviews included case reports and case series as primary studies [27,28,29,31,32,33,34,36,38,40,43], while other reviews also included prospective and retrospective studies [34,35,41,42]. Two SRs did not specify the type of primary studies included [26,39]. The number of total subjects included in each review was not always clarified. The diagnosis was related to different types of ameloblastoma: solid or multicystic [26,29,31,33,34,35,38,41,42,43], unicystic [29,30,31,32,33,34,35,38,39,40,41,42,43], desmoplastic ameloblastoma [27], peripheral ameloblastoma [28], adenoid ameloblastoma [32], sinonasal ameloblastoma [37]. All diagnoses were about primary ameloblastomas, while only one study also considered the recurrent form [36].

The surgical procedures studied were radical treatments, such as marginal and segmental resection [26,29,31,33,34,36,38,39,41,43], segmental mandibulectomy [31,42,43], and maxillectomy [37,41], and conservative treatments, such as curettage [28,29,31,35,36,37,38,42,44,45], enucleation [26,28,29,32,33,34,35,36,37,39,40,41,42,43], marsupialization, and decompression [30,33,36,40,42]. Both treatments were associated with adjuvant procedures, like cryotherapy [26,29,33,34,39], radiotherapy [28,32,37], Carnoy’s solution [26,33,34,35,36,40,41], bone reconstruction [27,31,43]. Some SRs did not indicate any control group [30,31,38]. In most of the studies, the primary outcome was the recurrence rate. Other reported outcomes were post-operative complications and patient-centered outcomes.

### 3.3. Methodological Quality Results

The methodological quality of the included reviews, as measured with the AMSTAR-2, ranged from critically low (three studies) to high (eight studies). The most common critical weakness in the included reviews was the absence of clearly a priori established review methods and any significant deviations from the protocol (Table 3).

### 3.4. Clinical Results

Most authors agreed that radical surgery, as marginal or segmental resection, was more appropriate in reducing the recurrence rate of both multicystic and unicystic ameloblastomas in comparison with conservative treatments [26,29,31,33,34,36,39,41,42,43]. Only Netto and collaborators [38] compared two radical approaches and pointed out that the group that underwent marginal resection was 1.1-times more likely to present recurrence of the lesion compared to the group that underwent segmental resection. However, there was no statistically significant difference between groups in all studies included.

Conservative treatments, like enucleation, decompression, and marsupialization, were not considered as a definitive surgery, but they were useful only to lower the invasiveness of the second surgery [30]. Lesion reduction was generally considered insufficient for these techniques to be used as definitive therapies. Moreover, according to Anpalagana A. [28] and Seintou A. et al. [40], a more conservative approach, consisting of an excision with narrow margin of normal tissue, was found to be appropriate for treating peripheral ameloblastomas with a low recurrence rate.

In Hendra et al. 2019 [33], the pooled recurrence rate of solid/multicystic ameloblastomas following radical treatment was 8%, while conservative treatment caused recurrences in 41%. For unicystic ameloblastomas, these values were 3% and 21%, respectively.

Similarly, Almeida et al. [26] showed that the relative risk of recurrence was 3.15-fold greater when conservative treatment was performed on primary multicystic ameloblastoma in comparison to radical treatment.

In da Silva et al. [41], the pooled values pointed out that the recurrence rate after the conservative surgery is neither comparable nor lower than the radical surgery (*p* = 0.28).

Seintou [40] and Anand [27] were the only researchers who took into account the age of patients in order to choose the best surgical option. According to both, a conservative approach is preferred in the case of young patients as it offers a better-quality life (functional limitation, physical pain, psychological discomfort). Moreover, according to Anand, specific lesions of less than 3 mm had to be treated by curettage in young people.

De Campos [31] was the only researcher who discussed the impact of surgical treatment of ameloblastoma on the oral health-related quality of life and the surgery-related complications, highlighting that invasive surgical treatment was associated with a high risk of post-operative complications, such as infections, fracture of cortical bone, plate traumatizing oral tissues, and graft loss. Finally, some of the included SRs dealt with the absence of an additive benefit of Carnoy’s solution as an adjuvant in the surgical treatment of ameloblastomas [35,36].

### 3.5. Meta-Analysis Results

The results of the meta-analysis are presented in Table 4. The primary outcome assessed was the recurrence rate. Only four studies included this in data synthesis [26,33,41,42], as they provided raw data on the recurrence rates for each study included. The meta-analysis revealed a significant combined effect size (RR = 3.01, 95% CI [2.02, 4.51], *p* < 0.001) with low heterogeneity (I^2^ < 50%) and a 95% prediction interval (PI) that did not include the value of 1. The test for excess statistical significance was not statistically significant (*p* = 0.177); however, Egger’s regression test indicated evidence of significant publication bias (*p* = 0.03).

## 4. Discussion

The aim of this overview was to summarize findings from systematic reviews (SRs) and meta-analyses on the radical or conservative treatment of ameloblastoma, to evaluate the methodological quality of the included SRs and discuss the clinical management. Based on the results of the current overview, we confirmed the intuitive concept that a radical approach leads to a lower recurrence rate. However, consideration of the post-operative complications and quality of life may be considered when the tumor affects young people or compromised patients. The recurrence rate depends not only on the surgical treatment but also on multiple other factors, like type of tumor, histological variants, surgical ability, and instruments used. It has been established that multicystic ameloblastoma exhibits a significantly higher recurrence rate compared to unicystic ameloblastoma [44]. Despite this, conservative treatment remains the primary approach for managing unicystic ameloblastoma [45,46]. Histological variants have previously been regarded as different types of ameloblastoma [47], each exhibiting different recurrence rates [48]. Despite these surgery-related risks, factors like the patient’s age, the anatomical location and size of the lesion, and its histological diagnosis should be considered in treatment planning to achieve a better prognosis. In this overview, only a few studies reported histological findings, making the data on recurrence rates not entirely comparable. However, the 2022 classification consolidated these variants into a single entity known as conventional ameloblastoma, potentially overcoming any selection bias. Regarding the surgical ability and instruments used, fully enucleating the lesion and removing all the possible tumor extensions still represent the major clinical challenge, especially for tumors located in proximity to important anatomic structures. A study by Troiano et al. revealed a lower rate of relapse at 5 years’ follow-up for patients treated with piezo surgery compared to conventional peripheral osteotomy in the treatment of conventional ameloblastoma located in proximity to the nervous alveolar bundle [49]. This method ensures highly effective hard tissue cutting and does not harm soft tissues, reporting lower post-operative complications [50]. The AMSTAR scale is a validated tool for assessing the methodological quality of systematic reviews (SRs). AMSTAR-2, developed to appraise both randomized and non-randomized healthcare intervention studies, includes 16 items and evaluates weaknesses in critical domains. This overview found that the methodological quality of reviews ranged from critically low to high, with the most common weakness being the absence of clearly established review methods and significant protocol deviations.

The meta-analysis revealed a significant combined effect size (RR = 3.01, 95% CI [2.02, 4.51], *p* < 0.001). This means that, on average, the recurrence rate is about three-times more likely in the conservative treatment group compared to the radical treatment group, and this result is statistically significant. The heterogeneity in this meta-analysis is low (I^2^ < 50%). Low heterogeneity suggests that the studies included in the meta-analysis are relatively consistent in their findings, and the combined effect size is a reliable estimate of the true effect. Another finding suggests that the 95% prediction interval (PI) did not include the value of 1. This is important because the prediction interval provides a range within which the effect size of a future study is expected to fall. Since the PI does not include 1, it suggests that even a new study is likely to find a similar positive effect, reinforcing the robustness of the findings.

The test for excess statistical significance did not show statistical significance (*p* = 0.177). This means there is no strong evidence that the observed results were due to an excess of studies with statistically significant findings, which could indicate selective reporting or other biases. However, Egger’s regression test indicated significant publication bias (*p* = 0.03). This finding suggests that the meta-analysis results might be influenced by publication bias, which could inflate the combined effect size. In summary, the meta-analysis demonstrates a strong and significant combined effect with low heterogeneity, but the presence of publication bias should be considered when interpreting the results.

### 4.1. Clinical Management

The surgical plan of ameloblastoma is determined after thorough clinical and radiographical investigations and histological diagnosis. A CT scan is useful for evaluating tumor boundaries and planning resection margins. For cases with cortical perforation and soft tissue infiltration, marginal or segmental resection, including soft tissue removal, is recommended [51]. Moreover, teeth involved with the tumor should be removed to prevent recurrence within the periodontal ligament [52]. Together with radical treatment, a reconstruction is needed to rehabilitate the esthetics and function [53], especially in young patients. In the present overview, few data have been reported about the complications related to radical surgery. Based on the results of this overview and our experience, we recommend conservative treatment as the first-line approach for intraosseous ameloblastoma not involving soft tissue. However, given the expectation of a higher recurrence rate, it is advisable to reduce the interval between follow-up visits. Early detection of recurrences, which are typically small and surrounded by a large amount of normal bone, allows for management with radical resection. This approach reduces the risk of further recurrence and helps avoid severe cosmetic and functional issues [54].

### 4.2. Future Perspectives

As reported before, traditionally, the treatment for ameloblastoma has been surgical. However, advancements in molecular biology have opened new perspectives for targeted therapies, particularly focusing on genetic mutations associated with the disease [55].

One of the most significant developments in the understanding of ameloblastoma at the molecular level is the identification of mutations in the BRAF gene [56]. The BRAF V600E mutation, which is common in various cancers, has been detected in a significant proportion of ameloblastoma cases. This discovery has opened the way for the potential use of BRAF inhibitors in the treatment of this tumor, such as vemurafenib and dabrafenib. These inhibitors work by specifically targeting and inhibiting the activity of the mutated BRAF protein, thereby reducing cell proliferation and inducing tumor regression [57]. In a recent study by Mamat Yusof et al. [58], the BRAF V600E mutation had a high pooled prevalence of 70.49% in ameloblastoma. Furthermore, a significant meta-analysis association was reported for those younger than 54 years old and in the mandible. On the contrary, other factors, such as sex, histological variants, and recurrence, were insignificant among ameloblastoma cases with the BRAF V600E mutation. In a study by Singh et al. [59], within the BRAFv600e+ group, females showed a higher reported recurrence rate. However, not all ameloblastomas present the BRAF V600E mutation, so patient selection based on genetic profiling will be important to optimize treatment efficacy. Research into the long-term outcomes of patients treated with BRAF inhibitors is necessary. If BRAF inhibitors prove to be effective, they could potentially reduce the need for extensive surgical procedures, leading to less morbidity and better cosmetic and functional results for patients. However, further well-designed cohort studies are needed to verify the association of the BRAF V600E mutation in ameloblastoma before applying new medical interventions.

### 4.3. Strengths and Limitations

The strength of the present study is the use of a high-quality search method adhering to PRISMA guidelines and a robust quality evaluation method following AMSTAR-2 standards. However, the findings of the current study should be understood in the light of important limitations. Although a comprehensive search strategy was employed and complemented through extensive manual cross-reference searching for the identification of all relevant articles, it may still be possible that some grey literature was missed. Additionally, it should be noted that most of the current literature reported mainly retrospective studies and case report/case series. Further prospective, multicenter, controlled trials with rigorous reporting and analysis of results and long-term follow-up-period studies are encouraged as they are lacking. Encouraging such studies would significantly strengthen the evidence base for ameloblastoma treatment. Moreover, long-term follow-up data are scarce, making it challenging to assess treatment efficacy. Establishing standardized follow-up protocols would facilitate more accurate assessments.

## 5. Conclusions

The primary finding of this umbrella review is that radical treatments for ameloblastoma are associated with significantly lower recurrence rates compared to conservative treatments. This suggests that radical approaches may offer better long-term disease control. On the other hand, with regard to post-operative complications and esthetic and functional impairments, few results arise from the currently published SRs. For clinicians, this review underscores the importance of weighing the benefits of lower recurrence rates against the risks of adverse outcomes, including esthetic and functional impairments. Moreover, the current overview of SRs highlighted that the quality level of the published SRs was extremely variable, thus ranging from critically low to high. Therefore, researchers are encouraged to focus on high-quality, prospective studies that can provide more definitive evidence on the comparative effectiveness and safety of radical versus conservative treatments. Improved methodological rigor and standardized outcome measures will enhance the reliability of future research and guide clinical decision making. Moreover, advancements in molecular biology may open up new perspectives for targeted therapies, focusing on genetic mutations associated with this disease. Further prospective studies are needed to establish the best treatment choice and follow-up period.

## Figures and Tables

**Table 1:** Search strategy for each database.

Databases	Search Strategy
Pubmed	(“ameloblastoma” [All Fields]) AND (“surgical treatment”[All Fields] OR “conservative treatment” [All Fields] OR “demolitive treatment” [All Fields] OR “radical treatment” [All Fields] OR “invasive treatment” [All Fields] OR “enucleation” [All Fields] OR “marsupialization” [All Fields] OR “resection”[All Fields] OR “maxillectomy” [All Fields] OR “mandibulectomy” [All Fields] OR “retreatment” [All Fields] OR “recurrence” [All Fields] OR “recurrence rate” [All Fields]) AND (“systematic review”[Publication Type] OR “meta analysis” [Publication Type])
Scopus	TITLE-ABS-KEY (ameloblastoma) AND (TITLE-ABS-KEY (surgical AND treatment) OR TITLE-ABS-KEY (conservative AND treatment) OR TITLE-ABS-KEY (demolitive AND treatment) OR TITLE-ABS-KEY (radical AND treatment) OR TITLE-ABS-KEY (invasive AND treatment) OR TITLE-ABS-KEY (enucleation) OR TITLE-ABS-KEY (marsupialization) OR TITLE-ABS-KEY (resection) OR TITLE-ABS-KEY (maxillectomy) OR TITLE-ABS-KEY (mandibulectomy) OR TITLE-ABS-KEY (recurrence) OR TITLE-ABS-KEY (recurrence AND rate) OR TITLE-ABS-KEY (retreatment)) AND (LIMIT-TO (DOCTYPE, “re”))
Cochrane	(Surgical treatment of ameloblastoma):ti,ab,kw

**Table 2:** Study characteristics.

Author, Year of Publication, Country	Search Period	Databases	Study Design	Diagnosis	Intervention	Control	Quality Tool and Quality of the Studies	Outcomes	Results	Conclusions
Almeida Rde et al., 2016 Brazil [26]	Up to January 2014	Ovid Medline and Embase	SR of 7 studies	Primary multicystic ameloblastoma	Marginal and segmental resection	Enucleation, enucleation + Carnoy’s, curettage, curettage + cryotherapy, marsupialization + enucleation	Modified scale of the Agency for Healthcare Research and Quality. Low-moderate risk of bias	Recurrence rate	The relative risk of recurrence was 3.15-fold greater (55–90%) when conservative treatment was performed on primary multicystic ameloblastoma in comparison to radical treatment (15–25%). Therefore, the risk of recurrence of the marginal resection is lower than segmental one	Significant results favouring radical treatment with bone resection for primary multicystic ameloblastoma
Anand R. et al., 2017 India [27]	From 1987 to May 2017	PubMed, Medline, Scopus, Web of Science and Google Scholar	SR of 76 case reports and case series	Primary desmoplastic ameloblastoma	Resection, resection + bone graft	Curettage	Not reported	Recurrence rate, size of lesion, patient’s age	The duration of the recurrence ranged from 2 to 6 years. In most of cases lesions < 3 mm were treated by curettage. Curettage was chosen mainly for young people	The type of surgery depended on the size of lesion rather than patient’s age
Anpalagana A. et al., 2020 England [28]	Not reported	Medline, EMBASE, Ovid Evidence-Based Medicine	Structured review of 34 case report, 2 case series, 1 retrospective case review, 1 case study, 5 review, 1 systematic review	Primary peripheral ameloblastoma	Radical surgical excision	Conservative surgical excision, radiotherapy	Not reported	Recurrence rate, recurrence presentation time	Overall, recurrence rate from 9% to 20% for supraperiosteal excision. Recurrence presentation time varied from 2 months to 7 years	The management of peripheral ameloblastoma appears to favour conservative excision with narrow margins of normal tissue
Antonoglou G. N. et al., 2014 Finland [29]	Up to December 2013	Medline, Scopus, LILACS, BBO, IBECS, ISI Web of Knowledge, Cochrane Database	SR of 7 case series	Primary unicystic and solid or multicystic ameloblastoma lesions	Enucleation with peripheral ostectomy, marginal and segmental resection, resection + cryosurgery, resection with encompassing dentoalveolar	Enucleation, enucleation + curettage, enucleation + cryotherapy, marsupialization	Risk of bias was assessed by Quality Appraisal Tool for Case Series, Grade Approach. The risk of bias was moderate	Recurrence rate	The recurrence rate of ameloblastomas for the unicystic and solid or multicystic variants ranged from 0.2% to 12% and 0.8% to 38% respectively	Resection may be preferable in both unicystic and solid or multicystic ameloblastomas
Berretta L.M. et al., 2021 Brazil [30]	Not reported	Embase, LILACS, PubMed, Scopus, The Cochrane Library, and Web of Science. Google Scholar, ProQuest	SR of 31 studies of which 8 are about ameloblastomas	Primary unicystic ameloblastomas	Marsupialisation and/or decompression	Not reported	Joanna Briggs Institute Critical Appraisal Checklist: low: 70%, moderate: 50% to 69%, high: 49%	Radiographic reduction measures of unicystic ameloblastomas	No significant differences were found regarding relative and absolute speeds of reduction considering lesion types	Lesion reduction was generally considered insufficient for these techniques to be used as definitive therapies, although benefits concerning the diminished invasiveness of the secondary surgery were often proposed
de Campos W.G. et al., 2022 Brazil [31]	Up to February 2021	PubMed, Science Direct, LILACS, EMBASE, and Web of Science; Google Scholar	SR of 10 case series	Primary solid/multicystic or unicystic ameloblastoma	Segmental resection + bone reconstruction, segmental mandibulectomy + bone reconstruction	Not reported	The Joanna Briggs Institute Critical Appraisal Checklist for Case Series: low-moderate	Recurrence rate, complications, quality of life	Recurrence rate was 0.7%; main complications were infections, fracture of cortical bone and graft loss; Quality of life was impaired due to functional limitation, physical pain, psychological discomfort	In addition to decreasing recurrence rates, the complete rehabilitation of patients after radical treatment of ameloblastoma should be a primary objective
de Farias Morais H.G. et al., 2023 Brazil [32]	From July to August 2022	PubMed, Web of Science, Scopus, EMBASE, Cochrane	RS of 15 case reports/series	Primary adenoid ameloblastoma	Surgical resection, Surgical resection + radiotherapy with or without neck dissection	Enucleation	CARE guidelines showed a low-moderate risk of bias	Recurrence rate; clinical, radiographical and histopathological findings	Recurrence rate was 30%; Swelling, pain and paresthesia were observed in 53.3%, 13.3%, 10% of cases respectively; radiografically, a well-defined radiolucency in 33,4% of the cases was observed; histologically, adenoid ameloblastoma showed a cribriform areas and duct-like structure in in 93.3% and 100% of the cases, respectively.	The adoption of initial conserva-tive management make it difficult to determine whether adenoid ameloblastoma has a higher risk of recurrence or more aggressive biological behavior than conventional ameloblastomas
Hendra F.N., 2019 The Netherlands [33]	From January 1969 until March 2018	PubMed, Embase, Scopus, and Web of Science	RS of 20 case series	Pimary solid/multicystic and unicystic ameloblastoma	Segmental/marginal resection	Enucleation, enucleation + Carnoy’s solution, enucleation + curettage, enucleation after marsupialization, curettage, curettage + criotherapy	Quality Appraisal of Case Series Studies Checklist (QACSS)	Recurrence rate	Multicystic ameloblastoma showed a recurrence rate of 8% after radical treatment, while it was 41% after conservative treatment. Unicystic ameloblastoma showed 3% e 21%, respectively	Statistically significant differences were found in recurrence favoring radical treatment for both unicystic and solid/multicystic ameloblastoma
Hendra F.N., 2023 The Netherlands [34]	Up to August 2021	PubMed, ScienceDirect, Scopus and Web of Science	SR of 7 case series	Primary solid/multicystic ameloblastoma	Segmental/marginal resection	Enucleation, enucleation + curettage, curettage + criotherpay, enucleation + Carnoy’s solution	Risk of bias in non-randomized studies-of exposure (ROBINS-E) showed a medium risk of bias	Recurrence rate	Segmental resection ranked highest for reducing the recurrence rate followed by curettage with cryotherapy and marginal resection	Segmental resection seemed to be the most effective treatment approach for minimizing recurrence in solid/multicystic ameloblastoma patients
Lal B. et al., 2021, India [35]	From 1980 to March 2020	PubMed, Google Scholar, Semantic Scholar, and Cochrane Library	SR of 39 case reports/series, retrospective and prospective studies	Primary mulcisystic/unicystic ameloblastoma	Resection + curettage	Enucleation + peripheral osteotomy + curettage, enucleation + curettage Carnoy’s solution	Not reported	Recurrence rate	Unicystic ameloblastome showed a recurrence rate of 10.98%, while multicystic ameloblastoma 18.18%;	There was no strong evidence for the use of Carnoy’s solutions as an adjuvant in the surgical treatment of ameloblastoma
Lau S.L., 2006 China [36]	Not reported	PubMed and Ovid, Embase and Cochrane Library	RS of 6 retrospective studies	Primary and recurrent ameloblastoma	Marginal/segmental resection	Enucleation, enucleation + Carnoy’s, marsupialization, marsupialization + enucleation + curettage	Cochrane reviewers’ handbook Section showed a low risk of bias	Recurrence rate	Recurrence rate was 3.6% for resection, 30.5% for enucleation alone, 16% for enucleation followed by application of Carnoy’s solution and 18% for marsupialization with/without other treatment in a second phase	Jaw resection resulted in the lowest recurrence rate, followed by enucleation with application of Carnoy’s solution
Mehta V. et al., 2023, India [37]	From 1998 to 2022	PubMed, Embase, Scopus, Google Scholar	RS of 15 case reports/series	Sinonasal ameloblastoma	Resection, maxillectomy + radiotherapy, endoscopic turbinectomy and medical maxillectomy	Enucleation	CARE guidelines checklist showed a low-moderate risk of bias	Recurrence rates, complication, mortality	Recurrence rate was 21% after surgical excision in one study; no post-operative complication after treatment except that numbness of tooth in one study; 0% mortality rate in all cases	Sinonasal ameloblastoma has a better outcome in terms of recurrence and complications after conservative and radical treatment compared to gnathic ameloblastomas
Netto R. et al., 2023 Brazil [38]	Up to July 2022	PubMed, ScienceDirect, Web of Science, Scopus, Embase, Google Scholar	SR of 8 case series	Primary solid/multicystic ameloblastomas	Segmental/marginal resection	Not reported	Joanna Briggs Institute Critical Appraisal Checklist for Case Series showed a moderate risk of bias	Recurrence rate	Recurrence rate after marginal resection ranged from 15.6 to 100% after segmental resection ranged from 5.6% to 25.0%	There was not statistically significant difference between the two groups in all studies
Qiao X. et al., 2021 China [39]	Up to October 2020	PubMed, Medline, Cochrane Library, and Embase, Google Scholar	SR of 20 studies	Primary solid/mulcisystic and unicystic ameloblastoma	Marginal/segmental resection, segmental resection, resection with bone margin, enucleation + peripheral ostectomy	Enucleation + cryosurgery	Newcastle-Ottawa Scale (NOS) scale showed a high quality in five studies, moderate quality in fifty studies	Recurrence rate	Recurrence rates of 0.08 and 0.41 for patients using aggressive and conservative treatments, respectively	Aggressive treatment might lead to a lower recurrence rate than conservative treatment
Seintou A. et al., 2014 Switzerland [40]	From1992 to 2012	PubMed	RS of 25 case series	Primary unicystic ameloblastoma	Resection	Enucleation + curettage, decompression before enucleation, excision before enucleation, marsupialization before enucleation, enucleation + Carnoy’s solution	Not reported	Recurrence rate	Recurrence rate was 29.4% in all cases treated with enucleation or excision. Luminal unicystic ameloblastomas are less respond better to conservative treatment. Plexiform and mural types frequently result in recurrence	Conservative treatment appears to be preferable in the younger age groups as it offers better quality of life, but the recurrence rate remains high
Slusarenko da Silva Y. et al., 2018 Brazil [41]	Up to May 2017	PubMed, Web of Science, Scopus and Cochrane Library	RS of 7 restrospective observational case controls, retrospective observational case, prospective case series	Primary solid/multicystic ameloblastoma	Segmental/marginal resection, subtotal maxillectomy/maxillectomy, enucleation + peripheral ostectomy	Enucleation, Enucleation + Carnoy,	Joanna Briggs Institute showed a low risk of bias	Recurrence rate	Conservative surgery is neither comparable nor lower than the radical surgery (*p* = 0.28)	Conservative surgery does not reduce the recurrence rate as efficiently as radical surgery for primary ameloblastomas
Troiano G, 2016 Italy [42]	From January 2005 to September 2015	PubMed, Ovid, EMBASE and Web of Science	SR of 4 non-randomized observational restrospective cohorts	Solid/multicistic ameloblastomas	Segmental/marginal resection, emimandibulectomy, segmental resection of the mandible	Enucleation, curettage, marsupialization, decompression	Cochrane collaboration tool showed a medium/high quality	Recurrence rate	Recurrence rate was 40% for the conservative and 10% for the radical treatment	A lower possibility of recurrence after radical treatment of solid/multicystic ameloblastoma was found
Vidya Ajila, 2022 India [43]	Between 2010 and 2020	Pubmed	RS of 16 case studies	Solid/multicystic and unicystic ameloblastomas	Surgical resection, segmental resection, emimandibulectomy + bone reconstruction	Enucleation, enucleation + curettage, enucleation + peripheral osteotomy	Not reported	Recurrence rate	Recurrence rate after conservative treatment was 64.9% and after radical treatment was 12%.	Radical management is recommended for solid/multicystic ameloblastomas in order to decrease the recurrence rate

SR, Systematic Review; LILACS, Latin American and Caribbean Health Sciences Literature; BBO, Brazilian Board of Orthodontics and Facial Orthopedics; IBECS, The Spanish Bibliographic Index of Health Sciences.

**Table 3:** Quality assessment of the included systematic review.

	Almeida Rde. et al., 2016 [26]	Rahul Anand et al., 2017 [27]	Anpalagan A. et al., 2020 [28]	Antonoglou G.N. et al., 2014 [29]	Berretta L.M. et al., 2021 [30]	De Campos W.G. et al., 2022 [31]	de Farias Morais H.G. et al., 2023 [32]	Hendra F.N. et al., 2019 [33]	Hendra F.N. et al., 2023 [34]	B. Lal et al., 2021 [35]	Lau S.L. et al., 2006 [36]
Did the research questions and inclusion criteria for the review include the components of PICO?	N	N	N	Y	Y	Y	Y	N	Y	Y	N
Did the report of the review contain an explicit statement that the review methods were established prior to the conduct of the review and did the report justify any significant deviations from the protocol?	N	N	N	N	N	N	N	N	N	N	N
Did the review authors explain their selection of the study designs for inclusion in the review?	N	Y	Y	Y	N	Y	Y	Y	Y	Y	Y
Did the review authors use a comprehensive literature search strategy?	Y	Y	Y	Y	Y	Y	Y	Y	Y	Y	Y
Did the review authors perform study selection in duplicate?	Y	N	Y	Y	Y	NR	Y	Y	Y	Y	Y
Did the review authors perform data extraction in duplicate?	Y	N	Y	Y	Y	NR	Y	Y	Y	Y	Y
Did the review authors provide a list of excluded studies and justify the exclusions?	Y	N	N	PY	N	Y	PY	Y	Y	Y	Y
Did the review authors describe the included studies in adequate detail?	Y	Y	Y	Y	Y	Y	PY	Y	Y	Y	Y
Did the review authors use a satisfactory technique for assessing the risk of bias (RoB) in individual studies that were included in the review?	Y	N	NR	Y	Y	Y	Y	Y	Y	N	Y
Did the review authors report on the sources of funding for the studies included in the review?	Y	N	N	Y	Y	N	Y	Y	Y	N	N
If meta-analysis was performed did the review authors use appropriate methods for statistical combination of results?	Y	Nm	Nm	Y	Nm	Nm	Nm	Y	Y	Nm	Nm
If meta-analysis was performed did the review authors assess the potential impact of RoB in individual studies on the results of the meta-analysis or other evidence synthesis?	Y	Nm	Nm	Y	Nm	Nm	Nm	Y	Y	Nm	Nm
Did the review authors account for RoB in individual studies when interpreting/discussing the results of the review?	Y	N	N	Y	Y	Y	Y	Y	Y	N	Y
Did the review authors provide a satisfactory explanation for, and discussion of, any heterogeneity observed in the results of the review?	Y	Y	N	Y	Y	Y	Y	Y	Y	Y	Y
If they performed quantitative synthesis did the review authors carry out an adequate investigation of publication bias (small study bias) and discuss its likely impact on the results of the review?	N	Nm	Nm	N	Nm	Nm	Nm	Y	Y	Nm	Nm
Did the review authors report any potential sources of conflict of interest, including any funding they received for conducting the review?	Y	N	Y	Y	Y	Y	Y	Y	Y	Y	N
**Overall Quality Assessment**	**H**	**CL**	**CL**	**H**	**M**	**M**	**M**	**H**	**H**	**L**	**M**
					**Mehta V. et al., 2022 [37]**	**Rafael Netto et al., 2023 [38]**	**Xue Qiao et al., 2021 [39]**	**Seintau A. et al., 2014 [40]**	**da Silva Y.S. et al., 2018 [41]**	**Troiano G. et al., 2017 [42]**	**Vidya Ajila et al., 2021 [43]**
Did the research questions and inclusion criteria for the review include the components of PICO?					Y	Y	N	N	N	Y	N
Did the report of the review contain an explicit statement that the review methods were established prior to the conduct of the review and did the report justify any significant deviations from the protocol?					N	N	N	N	N	N	N
Did the review authors explain their selection of the study designs for inclusion in the review?					Y	Y	Y	Y	Y	Y	Y
Did the review authors use a comprehensive literature search strategy?					Y	Y	Y	PY	Y	Y	PY
Did the review authors perform study selection in duplicate?					Y	Y	Y	N	Y	Y	Y
Did the review authors perform data extraction in duplicate?					Y	Y	Y	N	Y	Y	Y
Did the review authors provide a list of excluded studies and justify the exclusions?					N	Y	N	N	Y	Y	N
Did the review authors describe the included studies in adequate detail?					Y	Y	Y	Y	Y	Y	Y
Did the review authors use a satisfactory technique for assessing the risk of bias (RoB) in individual studies that were included in the review?					Y	Y	Y	N	Y	Y	N
Did the review authors report on the sources of funding for the studies included in the review?					Y	N	Y	Y	N	N	Y
If meta-analysis was performed did the review authors use appropriate methods for statistical combination of results?					Nm	Y	Y	Nm	Y	Y	Nm
If meta-analysis was performed did the review authors assess the potential impact of RoB in individual studies on the results of the meta-analysis or other evidence synthesis?					Nm	Y	Y	Nm	Y	Y	Nm
Did the review authors account for RoB in individual studies when interpreting/discussing the results of the review?					Y	Y	Y	N	Y	Y	PY
Did the review authors provide a satisfactory explanation for, and discussion of, any heterogeneity observed in the results of the review?					Y	Y	Y	Y	Y	Y	Y
If they performed quantitative synthesis did the review authors carry out an adequate investigation of publication bias (small study bias) and discuss its likely impact on the results of the review?					Nm	Y	Y	Nm	N	Y	Nm
Did the review authors report any potential sources of conflict of interest, including any funding they received for conducting the review?					Y	Y	Y	Y	Y	N	Y
**Overall Quality Assessment**					**M**	**H**	**H**	**CL**	**H**	**H**	**L**

Y, Yes; N, No; PY, Partial Yes; Nm, No meta-analysis; L, Low; CL, Critically Low; M, moderate; H, High.

**Table 4:** Meta-analysis results.

Factor	n_Studies	Total_n	n_Cases	RR [95% CI]	*p*_Value	I2	95% PI	Egger_p	ESB_p
ConservativeVs.Radical	15	998	269	3.02 [2.02; 4.51]	<0.001	28%	[1.092, 8.352]	0.03	0.177

RR: (relative risk); CI: confidence interval; I2: I-square statistics; PI: prediction interval.

## Data Availability

The original contributions presented in this study are included in the article; further inquiries can be directed to the corresponding author.

## References

[1] Masthan K.M., Anitha N., Krupaa J., Manikkam S. (2015). Ameloblastoma. J. Pharm. Bioallied Sci..

[2] Ghai S. (2022). Ameloblastoma: An Updated Narrative Review of an Enigmatic Tumor. Cureus.

[3] Hendra F.N., Van Cann E.M., Helder M.N., Ruslin M., de Visscher J.G., Forouzanfar T., de Vet H.C.W. (2020). Global incidence and profile of ameloblastoma: A systematic review and meta-analysis. Oral Dis..

[4] El-Naggar A.K., Chan J.K.C., Takata T., Grandis J.R., Slootweg P.J. (2017). The fourth edition of the head and neck World Health Organization blue book: Editors’ perspectives. Hum. Pathol..

[5] Gasparro R., Calabria E., Coppola N., Marenzi G., Sammartino G., Aria M., Mignogna M.D., Adamo D. (2021). Sleep Disorders and Psychological Profile in Oral Cancer Survivors: A Case-Control Clinical Study. Cancers.

[6] Chandu A., Smith A.C., Rogers S.N. (2006). Health-related quality of life in oral cancer: A review. J. Oral Maxillofac. Surg..

[7] Vered M., Wright J.M. (2022). Update from the 5th Edition of the World Health Organization Classification of Head and Neck Tumors: Odontogenic and Maxillofacial Bone Tumours. Head Neck Pathol..

[8] Correa-Arzate L., Portilla-Robertson J., Ramírez-Jarquín J.O., Jacinto-Alemán L.F., Mejía-Velázquez C.P., Villanueva-Sánchez F.G., Rodríguez-Vázquez M. (2023). LRP5, SLC6A3, and SOX10 Expression in Conventional Ameloblastoma. Genes.

[9] Augustine D., Rao R.S., Surendra L., Patil S., Yoithapprabhunath T.R., Albogami S., Shamsuddin S., Basheer S.A., Sainudeen S. (2022). Histopathologic Feature of Hyalinization Predicts Recurrence of Conventional/Solid Multicystic Ameloblastomas. Diagnostics.

[10] Yang R., Lin X., Zhang W., Gokavarapu S., Lin C., Ren Z., Hu Y., Cao W., Ji T. (2024). Unicystic ameloblastoma: A retrospective study on recurrent factors from a single institute database. Oral Dis..

[11] Arora S. (2015). Unicystic Ameloblastoma: A Perception for the Cautious Interpretation of Radiographic and Histological Findings. J. Coll. Physician. Surg. Park.

[12] Ide F., Ito Y., Miyazaki Y., Nishimura M., Kusama K., Kikuchi K. (2020). A New Look at the History of Peripheral Ameloblastoma. Head Neck Pathol..

[13] AlAli A.M., Hawkins D., Glass S. (2024). Peripheral ameloblastoma underlying squamous cell papilloma after a third molar extraction. Oral Surg. Oral Med. Oral Radiol..

[14] Adeel M., Rajput M.S.A., Arain A.A., Baloch M., Khan M. (2018). Ameloblastoma: Management and Outcome. Cureus.

[15] Elo J.A., Tandon R., Allen C.N., Murray M.D. (2014). Hemimaxillectomy for desmoplastic ameloblastoma with immediate temporalis flap reconstruction. Oral Surg. Oral Med. Oral Pathol. Oral Radiol..

[16] Kitisubkanchana J., Reduwan N.H., Poomsawat S., Pornprasertsuk-Damrongsri S., Wongchuensoontorn C. (2021). Odontogenic keratocyst and ameloblastoma: Radiographic evaluation. Oral Radiol..

[17] Di Lauro A.E., Romeo G., Scotto F., Guadagno E., Gasparro R., Sammartino G. (2022). Odontogenic keratocystic can be misdiagnosed for a lateral periodontal cyst when the clinical and radiographical findings are similar. Minerva Dent. Oral Sci..

[18] Liu Z., Liu J., Zhou Z., Zhang Q., Wu H., Zhai G., Han J. (2021). Differential diagnosis of ameloblastoma and odontogenic keratocyst by machine learning of panoramic radiographs. Int. J. Comput. Assist. Radiol. Surg..

[19] Dandriyal R., Gupta A., Pant S., Baweja H.H. (2011). Surgical management of ameloblastoma: Conservative or radical approach. Natl. J. Maxillofac. Surg..

[20] Giraddi G.B., Arora K., Saifi A.M. (2017). Ameloblastoma: A retrospective analysis of 31 cases. J. Oral Biol. Craniofac. Res..

[21] Ruslin M., Hendra F.N., Vojdani A., Hardjosantoso D., Gazali M., Tajrin A., Wolff J., Forouzanfar T. (2018). The Epidemiology, treatment, and complication of ameloblastoma in East-Indonesia: 6 years retrospective study. Med. Oral Patol. Oral Cir. Bucal.

[22] Laino L., Cicciù M., Russo D., Cervino G. (2020). Surgical Strategies for Multicystic Ameloblastoma. J. Craniofac. Surg..

[23] Yang Z., Liang Q., Yang L., Zheng G.S., Zhang S.E., Lao X.M., Liang Y.J., Liao G.Q. (2018). Marsupialization of mandibular cystic ameloblastoma: Retrospective study of 7 years. Head Neck.

[24] Shea B.J., Reeves B.C., Wells G. (2017). AMSTAR 2: A critical appraisal tool for systematic reviews that include randomised or non-randomised studies of healthcare interventions, or both. Br. Med. J..

[25] Gosling C.J., Solanes A., Fusar-Poli P., Radua J. (2023). Metaumbrella: The first comprehensive suite to perform data analysis in umbrella reviews with stratification of the evidence. BMJ Ment. Health.

[26] Almeida Rde A., Andrade E.S., Barbalho J.C., Vajgel A., Vasconcelos B.C. (2016). Recurrence rate following treatment for primary multicystic ameloblastoma: Systematic review and meta-analysis. Int. J. Oral Maxillofac. Surg..

[27] Anand R., Sarode G.S., Sarode S.C., Reddy M., Unadkat H.V., Mushtaq S., Deshmukh R., Choudhary S., Gupta N., Ganjre A.P. (2018). Clinicopathological characteristics of desmoplastic ameloblastoma: A systematic review. J. Investig. Clin. Dent..

[28] Anpalagan A., Tzortzis A., Twigg J., Wotherspoon R., Chengot P., Kanatas A. (2021). Current practice in the management of peripheral ameloblastoma: A structured review. Br. J. Oral Maxillofac. Surg..

[29] Antonoglou G.N., Sándor G.K. (2015). Recurrence rates of intraosseous ameloblastomas of the jaws: A systematic review of conservative versus aggressive treatment approaches and meta-analysis of non-randomized studies. J. Craniomaxillofac. Surg..

[30] Berretta L.M., Melo G., Mello F.W., Lizio G., Rivero E.R.C. (2021). Effectiveness of marsupialisation and decompression on the reduction of cystic jaw lesions: A systematic review. Br. J. Oral Maxillofac. Surg..

[31] de Campos W.G., Alkmin Paiva G.L., Esteves C.V., Rocha A.C., Gomes P., Lemos Júnior C.A. (2022). Surgical Treatment of Ameloblastoma: How Does It Impact the Oral Health-Related Quality of Life? A Systematic Review. J. Oral Maxillofac. Surg..

[32] de Farias Morais H.G., Gonçalo R.I.C., de Oliveira Costa C.S., de Figueiredo Pires H., Mafra R.P., de Morais E.F., da Costa Miguel M.C., de Almeida Freitas R. (2023). A Systematic Review of Adenoid Ameloblastoma: A Newly Recognized Entity. Head Neck Pathol..

[33] Hendra F.N., Natsir Kalla D.S., Van Cann E.M., de Vet H.C.W., Helder M.N., Forouzanfar T. (2019). Radical vs conservative treatment of intraosseous ameloblastoma: Systematic review and meta-analysis. Oral Dis..

[34] Hendra F.N., Helder M.N., Ruslin M., Van Cann E.M., Forouzanfar T. (2023). A network meta-analysis assessing the effectiveness of various radical and conservative surgical approaches regarding recurrence in treating solid/multicystic ameloblastomas. Sci. Rep..

[35] Lal B., Kumar R.D., Alagarsamy R., Shanmuga Sundaram D., Bhutia O., Roychoudhury A. (2021). Role of Carnoy’s solution as treatment adjunct in jaw lesions other than odontogenic keratocyst: A systematic review. Br. J. Oral Maxillofac. Surg..

[36] Lau S.L., Samman N. (2006). Recurrence related to treatment modalities of unicystic ameloblastoma: A systematic review. Int. J. Oral Maxillofac. Surg..

[37] Mehta V., Sarode G.S., Obulareddy V.T., Sharma T., Kokane S., Cicciù M., Minervini G. (2023). Clinicopathologic Profile, Management and Outcome of Sinonasal Ameloblastoma—A Systematic Review. J. Clin. Med..

[38] Netto R., Peralta-Mamani M., de Freitas-Filho S.A., Moura L.L., Rubira C.M., Rubira-Bullen I.R. (2023). Segmental resection vs. partial resection on treating solid multicystic ameloblastomas of the jaws—Recurrence rates: A systematic review and meta-analysis. J. Clin. Exp. Dent..

[39] Qiao X., Shi J., Liu J., Liu J., Guo Y., Zhong M. (2021). Recurrence Rates of Intraosseous Ameloblastoma Cases with Conservative or Aggressive Treatment: A Systematic Review and Meta-Analysis. Front. Oncol..

[40] Seintou A., Martinelli-Kläy C.P., Lombardi T. (2014). Unicystic ameloblastoma in children: Systematic review of clinicopathological features and treatment outcomes. Int. J. Oral Maxillofac. Surg..

[41] Slusarenko da Silva Y., Tartaroti N.A., Sendyk D.I., Deboni M.C.Z., Naclério-Homem M.D.G. (2018). Is conservative surgery a better choice for the solid/multicystic ameloblastoma than radical surgery regarding recurrence? A systematic review. Oral Maxillofac. Surg..

[42] Troiano G., Dioguardi M., Cocco A., Laino L., Cervino G., Cicciu M., Ciavarella D., Lo Muzio L. (2017). Conservative vs Radical Approach for the Treatment of Solid/Multicystic Ameloblastoma: A Systematic Review and Meta-analysis of the Last Decade. Oral Health Prev. Dent..

[43] Vidya A., Shruthi H. (2022). Ameloblastomas vs recurrent ameloblastomas: A systematic review. J. Oral Med. Oral Surg..

[44] Effiom O.A., Ogundana O.M., Akinshipo A.O., Akintoye S.O. (2018). Ameloblastoma: Current etiopathological concepts and management. Oral Dis..

[45] Leite-Lima F., Martins-Chaves R.R., Fonseca F.P., Brennan P.A., de Castro W.H., Gomez R.S. (2023). A conservative approach for unicystic ameloblastoma: Retrospective clinic-pathologic analysis of 12 cases. J. Oral Pathol. Med..

[46] Titinchi F., Brennan P.A. (2022). Unicystic ameloblastoma: Analysis of surgical management and recurrence risk factors. Br. J. Oral Maxillofac. Surg..

[47] Goh Y.C., Siriwardena B.S.M.S., Tilakaratne W.M. (2021). Association of clinicopathological factors and treatment modalities in the recurrence of ameloblastoma: Analysis of 624 cases. J. Oral Pathol. Med..

[48] Gupta K., Chaturvedi T.P., Gupta J., Agrawal R. (2019). Cell proliferation proteins and aggressiveness of histological variants of ameloblastoma and keratocystic odontogenic tumor. Biotech. Histochem..

[49] Troiano G., Inghingolo A., Serpico R., Ciavarella D., Lo Muzio L., Cervino G., Cicciù M., Laino L. (2018). Rate of Relapse After Enucleation of Solid/Multicystic Ameloblastoma Followed by Piezoelectric or Conventional Peripheral Ostectomy. J. Craniofac. Surg..

[50] Rullo R., Festa V.M., Rullo F., Trosino O., Cerone V., Gasparro R., Laino L., Sammartino G. (2016). The Use of Piezosurgery in Genioplasty. J. Craniofac. Surg..

[51] De Silva I., Rozen W.M., Ramakrishnan A., Mirkazemi M., Baillieu C., Ptasznik R., Leong J. (2012). Achieving adequate margins in ameloblastoma resection: The role for intra-operative specimen imaging. Clinical report and systematic review. PLoS ONE.

[52] Abbate V., Togo G., Committeri U., Zarone F., Sammartino G., Valletta A., Elefante A., Califano L., Dell’Aversana Orabona G. (2023). Full Digital Workflow for Mandibular Ameloblastoma Management: Showcase for Technical Description. J. Clin. Med..

[53] Petrovic I.D., Migliacci J., Ganly I., Patel S., Xu B., Ghossein R., Huryn J., Shah J. (2018). Ameloblastomas of the mandible and maxilla. Ear Nose Throat J..

[54] Sammartino G., Zarrelli C., Urciuolo V., di Lauro A.E., di Lauro F., Santarelli A., Giannone N., Lo Muzio L. (2007). Effectiveness of a new decisional algorithm in managing mandibular ameloblastomas: A 10-years experience. Br. J. Oral Maxillofac. Surg..

[55] Yiannis C., Mascolo M., Mignogna M.D., Varricchio S., Natella V., De Rosa G., Lo Giudice R., Galletti C., Paolini R., Celentano A. (2021). Expression Profile of Stemness Markers CD138, Nestin and Alpha-SMA in Ameloblastic Tumours. Int. J. Environ. Res. Public Health.

[56] Ebeling M., Scheurer M., Sakkas A., Pietzka S., Schramm A., Wilde F. (2023). BRAF inhibitors in BRAF V600E-mutated ameloblastoma: Systematic review of rare cases in the literature. Med. Oncol..

[57] Proietti I., Skroza N., Michelini S., Mambrin A., Balduzzi V., Bernardini N., Marchesiello A., Tolino E., Volpe S., Maddalena P. (2020). BRAF Inhibitors: Molecular Targeting and Immunomodulatory Actions. Cancers.

[58] Mamat Yusof M.N., Ch’ng E.S., Radhiah Abdul Rahman N. (2022). BRAF V600E Mutation in Ameloblastoma: A Systematic Review and Meta-Analysis. Cancers.

[59] Singh A.K., Alagarsamy R., Chaulagain R., Singh A., Sapkota D., Thavaraj S., Singh R.P. (2023). Does BRAF mutation status and related clinicopathological factors affect the recurrence rate of ameloblastoma? A systematic review, meta-analysis and metaregression. J. Oral Pathol. Med..

